# Clinical Profile, Humanistic and Economic Burden of Paroxysmal Nocturnal Hemoglobinuria in Patients Treated With C5 Inhibitors

**DOI:** 10.1002/jha2.70248

**Published:** 2026-02-18

**Authors:** Shreyans Gandhi, Markus P. Radsak, Yasutaka Ueda, Maria‐Magdalena Balp, Anggie Wiyani, Olivier Somenzi, Josefin Snellman, Yasmin Taylor, Alice Simons, Jens Panse

**Affiliations:** ^1^ Department of Haematological Medicine King's College Hospital NHS Trust London UK; ^2^ Department of Internal Medicine VII DONAUISAR Klinikum Deggendorf Germany; ^3^ Department of Hematology and Oncology Osaka University Graduate School of Medicine Suita Japan; ^4^ Novartis Pharma AG Basel Switzerland; ^5^ Novartis Pharmaceuticals UK London UK; ^6^ Adelphi Real World Bollington UK; ^7^ Department of Haematology, Oncology, Haemostaseology and Stem Cell Transplantation University Hospital RWTH Aachen Aachen Germany

**Keywords:** anemia, burden of disease, complement 5 inhibitor (C5i), hemoglobin, paroxysmal nocturnal hemoglobinuria, real‐world data

## Abstract

**Objectives:**

To evaluate disease burden among paroxysmal nocturnal hemoglobinuria (PNH) patients prescribed C5 inhibitors (C5i).

**Methods:**

Data were drawn from the Adelphi Real World PNH Disease Specific Programme, a cross‐sectional survey of physicians and PNH patients in Australia, Canada, France, Germany, Italy, Spain, the United Kingdom, and Japan from January–December 2022. Physicians reported patient demographics, laboratory parameters, and treatments. Patients completed the EQ‐5D‐5L, Functional Assessment of Chronic Illness Therapy – Fatigue (FACIT‐Fatigue), and Work Productivity and Activity Impairment (WPAI) questionnaire. Analyses were descriptive.

**Results:**

Overall, 81 physicians reported on 288 patients receiving C5i. Median (IQR) age was 50.0 (38.3–65.0) years, 79.2% were white, and 54.5% were male. Median (IQR) C5i duration was 1.0 (0–3.0) years; 77.4% received eculizumab, 22.6% ravulizumab. At time of survey, median (IQR) hemoglobin (Hb) was 11.0 (9.9–12.0) g/dL, 73.5% of patients had Hb < 12 g/dL. Lactate dehydrogenase was >1.5× upper limit of normal for 16.7% of patients. Mean (SD) EQ‐5D‐5L was 0.76 (0.20), FACIT‐Fatigue was 36.1 (9.7), WPAI work impairment was 27.5% (22.3) and activity impairment was 35.3% (24.6).

**Conclusions:**

Despite C5i treatment, a notable proportion of patients remained anemic and reported impaired quality of life, indicating the need for novel, efficacious therapies.

## 1 Introduction

Paroxysmal nocturnal hemoglobinuria (PNH) is a rare, acquired blood disorder, characterized by complement‐mediated hemolysis. Patients with PNH have an acquired somatic mutation in the phosphatidylinositol glycan biosynthesis class A (PIGA) gene within hematopoietic stem cells, leading to a deficiency or absence of complement regulatory proteins, such as CD55 and CD59 [1]. The loss of these proteins on the surface of red blood cells, increases their susceptibility to complement‐mediated hemolysis [2]. The subsequent activation of leukocytes and platelets creates a favorable environment for thrombosis [2], which is associated with morbidity and mortality in patients with PNH [3]. Manifestations of PNH include clinical features of hemolysis, hemoglobinuria, and fatigue [4, 5].

The first treatments approved for PNH include complement 5 inhibitors (C5i), eculizumab and ravulizumab [6, 7]. Eculizumab, administered biweekly through intravenous infusion, has been shown in clinical trials to significantly reduce hemolysis, fatigue, the need for transfusions, the occurrence of thrombosis, and has improved long‐term survival in patients with PNH [6]. Ravulizumab offers similar efficacy and is administered intravenously every 8 weeks [8].

While C5i treatments have been demonstrated to improve outcomes for patients with PNH, such as improved organ function and reduced mortality [9], real‐world studies have highlighted suboptimal responses to treatment, with a relevant proportion of patients continuing to experience a significant burden of illness, including residual anemia and fatigue [10]. The persistence of symptoms can have a number of health consequences, including a decrease in overall and psychological wellbeing, as well as interference with daily activities and work [10]. As additional treatments for patients with PNH have been approved, either as first line therapies or for those with residual anemia despite treatment with C5i, it is important to further characterize the full burden of PNH in patients currently receiving C5i in real‐world settings.

The objective of this study was to gain a better understanding of the real‐world clinical, humanistic, and socioeconomic burden among patients with PNH treated with C5i, in a broad geographic setting.

## 2 Methods

### 2.1 Study Design and Database Description

This study was a secondary analysis of data using the Adelphi Real World PNH Disease Specific Programme (DSP), a cross‐sectional survey, with elements of retrospective data collection, of physicians and their consulting patients with PNH. The DSP was conducted in Australia, Canada, France, Germany, Italy, Japan, Spain, and the United Kingdom between January and December 2022. The DSP methodology has been previously described [11, 12], validated [13] and demonstrated to be representative and consistent over time [14]. The DSP comprises a physician‐completed patient survey and a patient self‐completed survey described below.

### 2.2 Physician‐Completed Patient Survey

Hematologists, oncologists and/or hematologist‐oncologists were eligible to participate, provided they were personally responsible for management and treatment decisions of patients with PNH. Physicians were recruited through publicly available lists, and the data collection setting was secondary hematology services (public or private hospitals, clinics, or offices).

Physicians completed surveys for up to their next 10 consecutively consulting patients with a confirmed diagnosis of PNH. Data analyzed from the surveys included: patient demographics, comorbidities, laboratory parameters such as hemoglobin, lactate dehydrogenase (LDH), reticulocyte count, and PNH clone size (defined as proportion of PNH cells) of which values at diagnosis and at survey were reported, treatment, number of thrombotic events and/or blood transfusions within the 12 months prior to time of survey. Outcomes captured for a 12‐month period prior to survey may include events that occurred prior to treatment initiation for patients who initiated C5i therapy within that period. For laboratory parameters, the last value closest to the time of survey was used as a proxy for “value at time of survey”. For the 12 months prior to survey, physicians reported healthcare resource utilization including healthcare professionals (HCPs) involved in patient management and number of consultations, number of hospitalizations, reasons for hospitalizations, and duration of stay. Completion of the survey was undertaken through consultation of existing patient clinical records, as well as the judgment and diagnostic skills of the respondent physician, which is consistent with decisions made in routine clinical practice to reflect real world settings.

### 2.3 Patient Self‐Completed Survey

Patients for whom the physician completed a survey were invited to voluntarily complete a patient self‐complete questionnaire. Patients were eligible for inclusion if they were ≥ 17 years old and had a physician‐confirmed diagnosis of PNH. The questionnaire comprised self‐reported sociodemographics, symptoms, and patient‐reported outcome measures: EQ‐5D‐5L and Visual Analog Scale (EQ‐VAS), the Medical Outcomes Study 36‐Item Short Form Survey version 1 (SF‐36), the Functional Assessment of Chronic Illness Therapy – Fatigue (FACIT‐Fatigue), and the Work Productivity and Activity Impairment (WPAI) questionnaire.

#### 2.3.1 EQ‐5D‐5L/EQ‐Visual Analogue Scale

The EQ‐5D‐5L measures generic health‐related quality of life (HRQoL) across five dimensions: mobility, self‐care, usual activities, pain/discomfort, and anxiety/depression [15, 16]. Per dimension there are five levels (5L) of response, ranging from no problems to extreme problems. Utility scores were calculated by mapping the 5L descriptive system data onto the 3L using the Hernandez‐Alava UK value set [17, 18], with scores ranging from 1 (*full health*) to 0 (*health state equivalent to dead*). The EQ‐VAS assesses overall health on the day of completion on a scale of 0 (*worst imaginable health*) to 100 (*best imaginable health*).

#### 2.3.2 SF‐36

The SF‐36 assesses HRQoL across an eight‐scale profile of functional health and wellbeing over the prior 4 weeks, together with two psychometrically based physical and mental health components [19]. Physical component scores (PCS) and mental component scores (MCS) range from 0 to 100, with lower scores indicating poorer outcomes. The PCS and MCS can be compared with normative data for the general population, standardized to have a mean of 50 and standard deviation (SD) of 10 [20, 21].

#### 2.3.3 FACIT‐Fatigue

The FACIT‐Fatigue questionnaire evaluates fatigue in patients with chronic illnesses such as PNH, over the 7 days prior to survey completion [22]. It consists of 13 items, scored from 0 to 4, with a maximum possible score of 52 points, and higher scores indicating less fatigue. General population norms have been previously published [23].

#### 2.3.4 Work Productivity and Activity Impairment

The WPAI measures the degree of work and activity impairment over the 7 days prior to survey. The WPAI consists of four domain scores: absenteeism (work time missed), presenteeism (impairment at work/reduced on‐the‐job effectiveness), work productivity loss (overall work impairment/absenteeism plus presenteeism), and activity impairment [24]. Scores range from 0% to 100%, with higher scores indicating greater impairment.

### 2.4 Data Analysis

This analysis focused on patients prescribed C5i at the time of survey, and those who were on C5i for at least 12 months. All analyses were descriptive and pooled. Means with SDs, and medians with interquartile ranges (IQRs) were calculated for continuous variables, and frequency counts and percentages for categorical variables. All analyses were conducted in UNICOM Intelligence Reporter version 7.5.1 (UNICOM Systems 2021). Missing data were not imputed; therefore, the base of patients for analysis could vary from variable to variable and is reported as appropriate.

## 3 Results

### 3.1 Physician‐Reported Data

#### 3.1.1 Demographic and General Clinical Characteristics of C5i‐Treated Patients

A total of 81 physicians reported data for 378 patients with PNH from Australia, Canada, Europe (France, Germany, Italy, Spain, and the United Kingdom), and Japan. At the time of survey, 288 (76.2%) patients were prescribed C5i (C5i‐treated), and of these, 163 (56.6%) had been prescribed C5i for at least 12 months (C5i ≥ 12 months; Table 1).

**TABLE 1:** Physician‐reported demographics and clinical characteristics of C5i‐treated patients.

	C5i‐treated patients	C5i ≥ 12 months
	*n* = 288	*n* = 163
**Geographic distribution, *n* (%)**		
Germany	66 (22.9)	25 (15.3)
France	43 (14.9)	34 (20.9)
Japan	39 (13.5)	31 (19.0)
Italy	34 (11.8)	21 (12.9)
Canada	27 (9.4)	11 (6.7)
UK	27 (9.4)	14 (8.6)
Australia	26 (9.0)	13 (8.0)
Spain	26 (9.0)	14 (8.6)
**Age (years), median (IQR)**	50.0 (38.3, 65.0)	52.0 (40.0, 67.0)
**BMI (kg/m^2^), mean (SD)**	24.0 (3.2)	23.9 (3.1)
**Male, *n* (%)**	157 (54.5)	86 (52.8)
**Ethnicity, *n* (%)**		
White	228 (79.2)	120 (73.6)
Japanese	38 (13.2)	30 (18.4)
Hispanic/Latino	7 (2.4)	5 (3.1)
Other	15 (5.2)	8 (4.9)
**Employment status, *n* (%)**		
Working full‐time	100 (34.7)	61 (37.4)
Retired	59 (20.5)	38 (23.3)
Working part‐time	51 (17.7)	28 (17.2)
Homemaker	30 (10.4)	17 (10.4)
Unemployed	21 (7.3)	11 (6.7)
On long‐term sick leave	18 (6.3)	3 (1.8)
Student	9 (3.1)	5 (3.1)
**Time since confirmatory diagnosis of PNH (years)**		
Patients with data, *n*	284	162
Median (IQR)	2.6 (1.0, 5.5)	4.1 (2.2, 7.2)

Abbreviations: BMI, body mass index; C5i, C5 inhibitor; IQR, interquartile range; SD, standard deviation; UK, United Kingdom.

Among C5i‐treated patients (*n* = 288), median (IQR) age was 50.0 (38.3, 65.0) years, 79.2% were white, 54.5% were male, and 52.4% were employed at the time of survey (Table 1). The median (IQR) time since confirmatory diagnosis of PNH was 2.6 (1.0, 5.5) years. The most common comorbidities reported by physicians at the time of survey were hypertension (21.5%), anxiety (12.2%), aplastic anemia (11.5%), depression (10.4%), and diabetes (10.4%), most of which were present prior to the onset of PNH symptoms (Table S1).

#### 3.1.2 Clinical Profile of C5i‐Treated Patients

**FIGURE 1:**
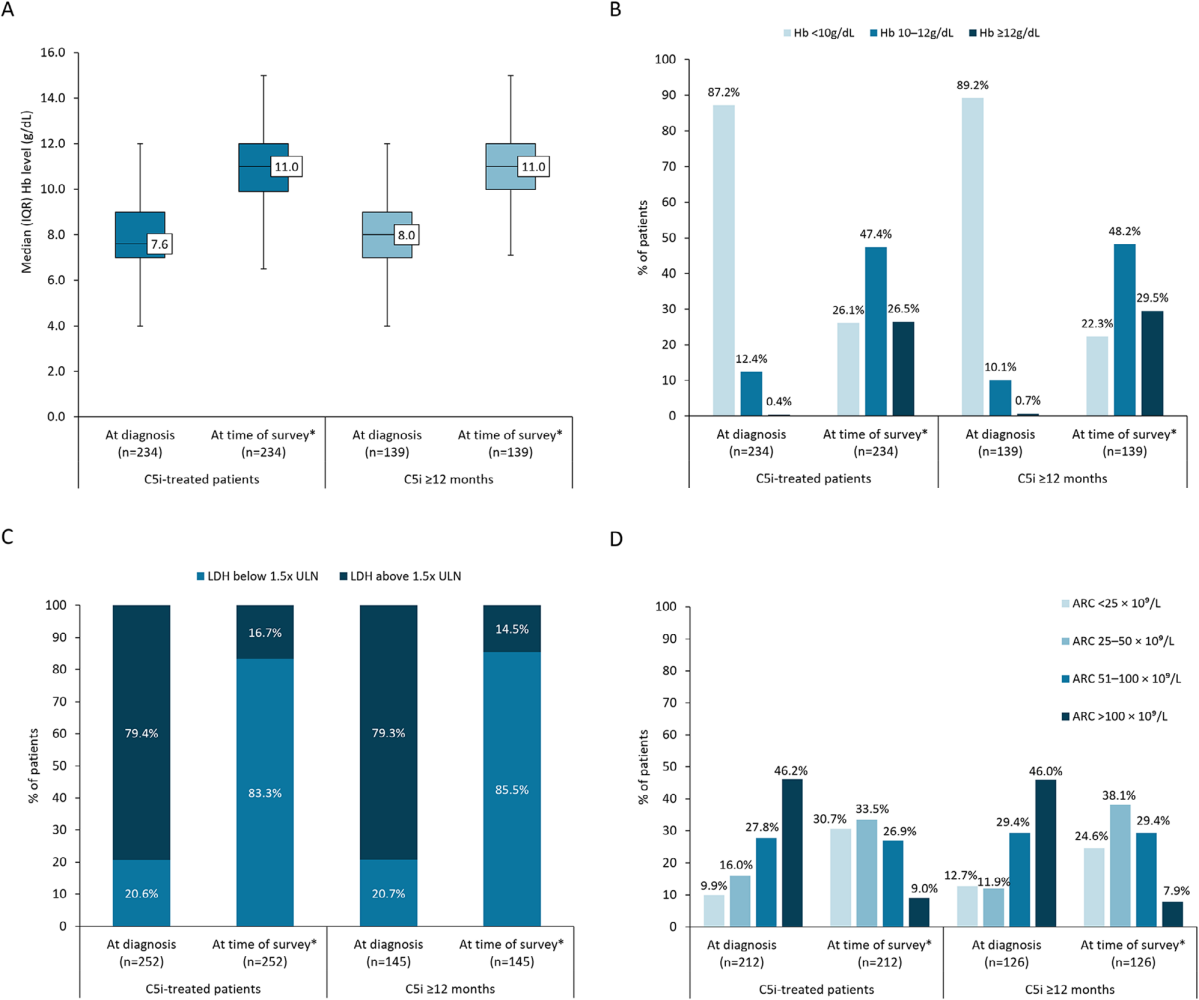
Clinical profile of C5i‐treated PNH patients. (A) Median (IQR) hemoglobin levels (g/dL) at diagnosis and at time of survey. (B) Percentage of patients with hemoglobin levels < 10 g/dL, 10–12 g/dL, and ≥12 g/dL at diagnosis and at time of survey. (C) Lactate dehydrogenase levels (U/L) 1.5 × ULN at diagnosis and at time of survey. (D) Absolute reticulocyte count (ARC) at diagnosis and at time of survey. *The last value closest to the time of survey was used as a proxy for value “at time of survey”. ARC, absolute reticulocyte count; Hb, hemoglobin; IQR, interquartile range; LDH, lactate dehydrogenase; ULN (defined as 250 U/L), upper limit of normal.

Median (IQR) hemoglobin level was 7.6 (7.0, 9.0) g/dL at the time of PNH diagnosis, and 11.0 (9.9, 12.0) g/dL at the time of survey (Figure 1A). At the time of survey, only 26.5% of C5i‐treated patients had achieved normalized hemoglobin levels (≥ 12g/dL), with 73.5% remaining anemic (Figure 1B). At the time of diagnosis, most C5i‐treated patients (79.4%) had LDH levels above 1.5× upper limit of normal (ULN), 46.2% had an absolute reticulocyte count > 100 × 10^9^/L and mean (SD) PNH clone size was 50.9% (26.9). At the time of survey, 16.7% of patients had LDH > 1.5 × ULN, 9.0% had absolute reticulocyte count > 100 × 10^9^/L and clone size was 37.4% (30.5; Figure 1C,D). The results for the clinical profile of patients treated with C5i ≥ 12 months can be found in Figure 1.

Overall (*n* = 279), 10.8% of C5i‐treated patients and 6.3% of those treated with C5i ≥ 12 months (*n* = 159), had experienced one or more thrombotic events in the 12 months prior to survey, with a mean (SD) number of events of 1.4 (1.0) and 1.4 (0.7), respectively.

#### 3.1.3 Disease Management of C5i‐Treated Patients

Patients had been receiving C5i for a median (IQR) of 1.0 (0.0, 3.0) years (Table 2). Of all C5i‐treated patients, 77.4% were prescribed eculizumab and 22.6% were prescribed ravulizumab. A total of 179 (62.2%) patients received concomitant treatments, which included dietary supplements or treatment for deficiencies (62.6%), anticoagulants (58.1%), and long‐term antibiotic therapy (28.5%). Dietary supplements and treatment for deficiencies included folate/folic acid (52.5%), vitamin B12 supplements (20.7%), and iron supplements (19.6%). Where PNH‐related blood transfusion data were available (*n* = 266), 40.6% had received one or more transfusions in the previous 12 months, and 12.8% had received five or more. Of these patients, 20.4% were considered transfusion dependent by their physicians. For patients who had been prescribed C5i therapy for ≥ 12 months (*n* = 158), 27.2% received at least one transfusion, and 8.2% received five or more transfusions during this period.

**TABLE 2:** Physician‐reported disease management of C5i‐treated patients.

	C5i‐treated patients	C5i ≥ 12 months
	*n* = 288	*n* = 163
**Duration of C5i treatment (years)**		
Patients with data, *n*	272	163
Median (IQR)	1.0 (0, 3.0)	2.0 (1.0, 4.0)
**C5i treatment at time of survey**		
Patients with data, *n*	288	163
Eculizumab, *n* (%)	223 (77.4)	132 (81.0)
Ravulizumab, *n* (%)	65 (22.6)	31 (19.0)
**Concomitant therapies to C5i** ^a^ **(Top 5 treatment classes**)		
Patients with data, *n*	179	93
Dietary supplements/treatment for deficiencies, *n* (%)	112 (62.6)	53 (57.0)
Anticoagulants, *n* (%)	104 (58.1)	51 (54.8)
Long‐term antibiotic therapy, *n* (%)	51 (28.5)	27 (29.0)
Analgesics, *n* (%)	39 (21.8)	11 (11.8)
Corticosteroids, *n* (%)	32 (17.9)	12 (12.9)
**Number of PNH‐related blood transfusions in the 12 months prior to survey**		
Patients with data, *n*	266	158
Mean (SD)	1.8 (3.5)	1.3 (3.4)
At least one transfusion, *n* (%)	108 (40.6)	43 (27.2)
Of those who had at least one transfusion, *n*	108	43
Mean (SD)	4.4 (4.3)	4.6 (5.1)
**Transfusion‐dependent (physician perceived)**		
Patients with data, *n*	108	43
Yes, *n* (%)	22 (20.4)	13 (30.2)

Abbreviations: C5i, C5 inhibitor; IQR, interquartile range; PNH, paroxysmal nocturnal hemoglobinuria; SD, standard deviation.

^a^ The top five treatment classes received concomitantly with C5i are presented. Treatments are not mutually exclusive, which means patients may have received multiple treatment classes concurrently.

#### 3.1.4 Healthcare Resource Utilization Among C5i‐Treated Patients

The most frequent HCPs reported to have been involved in the management of C5i‐treated patients within the 12 months prior survey were: hematologists (61.5%), hematologist–oncologists (36.5%), and general practitioners or primary care physicians (26.7%; Table 3). The mean (SD) number of visits to any HCP in the 12 months prior to survey was 11.3 (9.9) visits for C5i‐treated patients, and 11.5 (10.3) visits for those prescribed C5i ≥ 12 months (Table 3).

**TABLE 3:** Physician‐reported healthcare resource utilization of C5i‐treated patients.

	C5i‐treated patients	C5i ≥ 12 months
	*n* = 288	*n* = 163
**HCPs involved in management of PNH at time of survey (Top 5)** ^a^		
Patients with data, *n*	288	163
Hematologist, *n* (%)	177 (61.5)	99 (60.7)
Hematologist–oncologist, *n* (%)	105 (36.5)	62 (38.0)
General practitioner/primary care physician, *n* (%)	77 (26.7)	38 (23.3)
Specialist nurse, *n* (%)	32 (11.1)	17 (10.4)
Nephrologist, *n* (%)	23 (8.0)	6 (3.7)
**Number of visits to any HCP in the 12 months prior to survey**		
Patients with data, *n*	288	163
Mean (SD)	11.3 (9.9)	11.5 (10.3)
**Number of hospitalizations in the 12 months prior to survey**		
Patients with data, *n*	274	156
≥ 1 hospitalization, *n* (%)	49 (17.9)	19 (12.2)
**Number of PNH related hospitalizations in the 12 months prior to survey, for patients with ≥ 1 hospitalization**		
Patients with data, *n*	49	19
Mean (SD)	1.9 (1.9)	1.4 (0.8)
**Reason for most recent hospitalization**		
Patients with data, *n*	49	19
Infection, *n* (%)	16 (32.7)	8 (42.1)
Severe fatigue, *n* (%)	15 (30.6)	3 (15.8)
Other, *n* (%)	7 (14.3)	5 (26.3)
Thrombotic event, *n* (%)	6 (12.2)	2 (10.5)
Kidney failure, *n* (%)	5 (10.2)	1 (5.3)
**Most recent hospitalization via ER**		
Patients with data, *n*	48	19
Yes, *n* (%)	35 (72.9)	13 (68.4)
No, *n* (%)	13 (27.1)	6 (31.6)
**Most recent hospital stay**		
Patients with data, *n*	48	19
Overnight stay, *n* (%)	41 (85.4)	18 (94.7)
Day case, *n* (%)	7 (14.6)	1 (5.3)
**Length of most recent hospital stay (number of nights)**		
Patients with data, *n*	41	18
Mean (SD)	7.3 (6.9)	5.4 (3.9)
**Blood transfusions required (most recent hospitalization)**		
Patients with data, *n*	45	19
Yes, *n* (%)	27 (60.0)	10 (52.6)
No, *n* (%)	18 (40.0)	9 (47.4)

Abbreviations: C5i, C5 inhibitor; ER, emergency room; HCP, healthcare professional; SD, standard deviation

aHCPs involved in patient management were a multiple‐choice question; options were not mutually exclusive and patients may have received care from multidisciplinary team of HCPs.

Within the 12 months prior to survey, 17.9% of C5i‐treated patients and 12.2% of C5i ≥ 12 months had at least one hospitalization (Table 3). The main reasons for the most recent hospitalization for each subgroup included infection (32.7% and 42.1%) and severe fatigue (30.6% and 15.8%). For the most recent hospitalization, the majority were admitted via an emergency room (72.9% and 68.4%), and most patients required overnight stay (85.4% and 94.7%), for a mean (SD) of 7.3 (6.9) and 5.4 (3.9) nights, respectively.

### 3.2 Patient‐Reported Data

#### 3.2.1 Sociodemographic and Symptoms Among C5i‐Treated Patients at Time of Survey

Of the total of 146 patients who returned the questionnaire, 104 (71.2%) were receiving C5i at the time of survey. Of these (*n* = 104), 50.0% were male, with a median (IQR) age of 50.0 (38.3, 64.0) years (Table 4). Less than half of patients (42.7%) were in employment, 17.5% were retired, and 5.8% were studying. Patients reported a median (IQR) of 1.5 (0.0, 3.0) days off work or study in the previous 3 months due to their PNH. The most common symptoms reported by C5i‐treated patients included tiredness (91.3%), anemia (78.8%), shortness of breath (53.8%), headaches (49.0%), and anxiety (39.4%).

**TABLE 4:** Patient‐reported sociodemographic of C5i‐treated patients.

	C5i‐treated patients
**Total number of patients returning a self‐completed survey**	***n* = 104**
**Age (years), median (IQR)**	50.0 (38.3, 64.0)
**Male, *n* (%)**	52 (50.0)
**Time from symptom onset to diagnosis (weeks)**	
Patients with data, *n*	100
Median (IQR)	4.0 (3.0, 12.0)
**Current employment status**	
Patients with data, *n*	103
Working full‐time	29 (28.2)
Retired	18 (17.5)
Homemaker	17 (16.5)
Working part‐time	15 (14.6)
On long‐term sick leave	12 (11.7)
Unemployed	6 (5.8)
Student	6 (5.8)
**Days off work/study in the previous 3 months due to PNH**	
Patients with data, *n*	47
Median (IQR)	1.5 (0.0, 3.0)
**Impact of PNH on employment/career (past or present) (Top 5)**	
Patients with data, *n*	93
No impact (or not applicable), *n* (%)	42 (45.2)
Stopped working, *n* (%)	18 (19.4)
Changed to part‐time/reduced working hours, *n* (%)	13 (14.0)
Needed flexible working hours, *n* (%)	11 (11.8)
Difficulty concentrating at work, *n* (%)	9 (9.7)
Needed/had unplanned time off work, *n* (%)	7 (7.5)

Abbreviations: C5i, C5 inhibitor; IQR, interquartile range; PNH, paroxysmal nocturnal hemoglobinuria; SD, standard deviation

#### 3.2.2 Impact of PNH on Patient HRQoL and Wellbeing Among C5i‐Treated Patients

**FIGURE 2:**
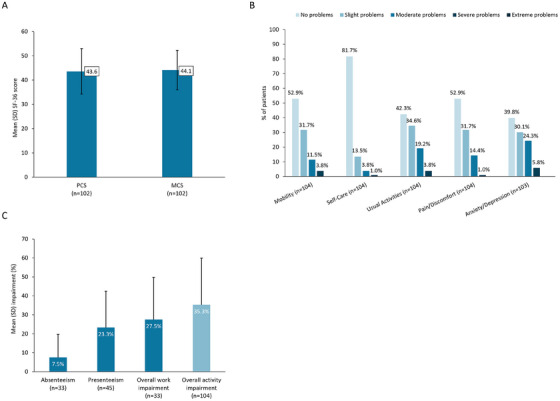
Patient‐reported impact of PNH on HRQoL and work and productivity in patients receiving C5i. (A) Mean (SD) SF‐36 score on the PCS and MCS. (B) Patient‐reported health state across the five dimensions of the EQ‐5D. (C) Mean (SD) impairment (%) for absenteeism, presenteeism, overall work impairment, and overall activity impairment, reported using the WPAI questionnaire. C5i, C5 inhibitor; EQ‐5D, EuroQol five dimensions; HRQoL, health‐related quality of life; MCS, mental component scores; PCS, physical component scores; SD, standard deviation; SF‐36, Short Form Health Survey; WPAI, work productivity and impairment.

Patient‐reported health status on the SF‐36 showed impairment of physical and mental status, reflected by the mean (SD) PCS and MCS scores of 43.6 (9.4) and 44.1 (8.1) (Figure 2A). The mean (SD) EQ‐5D VAS score was 70.0 (15.7) and the mean EQ‐5D utility score was 0.76 (0.20). Domain scores showed that patients experienced moderate–extreme problems across all EQ‐5D domains, including mobility (15.4%), self‐care (4.8%), daily activities (23.1%), pain or discomfort (15.4%), and anxiety or depression (30.1%; Figure 2B). The mean (SD) FACIT‐Fatigue score was 36.1 (9.7).

#### 3.2.3 Impact of PNH on Work and Activities Among C5i‐Treated Patients

Patients reported their PNH to have, at any time, caused them to stop working (19.4%), change their working hours (14.0%) or require flexible working hours (11.8%; Table 4). Using the WPAI, C5i‐treated patients who were employed at the time of survey had a mean (SD) percentage impairment of 7.5 (12.1) for absenteeism, 23.3 (19.2) for presenteeism, and an overall work impairment of 27.5 (22.3; Figure 2C). Overall percentage activity impairment, which includes all respondents, was 35.3 (24.6).

## 4 Discussion

In this real‐world study, we described demographics, clinical characteristics and disease burden of patients with PNH prescribed C5i across Australia, Canada, France, Germany, Italy, Spain, the United Kingdom, and Japan. Expected outcomes in the course of treatment include reduced intravascular hemolysis (as measured by LDH) and fatigue, improvement of Hb levels with expected normalization, improved HRQoL, and reduction in thrombotic events [25]. However, in this study, we found that the majority of patients remained anemic (Hb levels ≤ 12 g/dL) despite treatment with C5i, which demonstrates an important unmet clinical need. In addition, 40.6% of all patients receiving C5i and 27.2% of those receiving C5i for ≥ 12 months required at least one blood transfusion in the 12 months prior to survey, with 20.4% and 30.2%, respectively, considered to be transfusion‐dependent by their physician. A notable proportion of patients continued to receive anticoagulant therapy alongside C5i, including those treated for ≥ 12 months; however, the clinical rationale for this could not be assessed in this survey. These findings highlight the ongoing clinical burden of PNH, emphasizing the need for optimized disease management to address persistent anemia and minimize transfusion dependence.

Within our analysis, C5i‐treated patients reported a mean FACIT‐Fatigue score of 36.1, which is below the published general population mean (SD) score of 43.5 (8.3) [23]. Fatigue has previously been shown to significantly impact the overall wellbeing of patients with PNH, whereby higher fatigue severity of those receiving complement inhibitors is associated with worse HRQoL and functional impairment [26]. Further to this, patients in the present study had a mean EQ‐5D utility score of 0.76. General population norms have been reported as 0.88 in Germany [27] and 0.91 in France [28], which may suggest that patients in our study were experiencing impairment in HRQoL. As the EQ‐5D is a generic instrument, it may lack sensitivity to the specific symptoms and challenges of PNH, potentially underestimating HRQoL impairment. PNH led many patients to make changes to their employment, with 19.4% reporting that they had to stop working altogether. Findings from the WPAI questionnaire indicated that patients had a relevant overall work (27.5%) and activity impairment (35.3%). This means that patients could work at less than three quarters (72.5%) of their capacity or perform general activities at 64.7% capacity. This aligns with a previous study which found similar overall work (37%) and activity (39%) impairment in patients receiving eculizumab and ravulizumab [10]. Collectively, these findings emphasize that patients continue to experience disease burden, with substantial impacts on employment and productivity, despite receiving standard of care treatment.

In addition to the impact on HRQoL and productivity, we found that nearly 20% required hospitalization in the 12 months prior to survey, predominantly due to infection or fatigue. Among those receiving C5i, who had been hospitalized during this time, physicians reported an average of 1.9 PNH‐related hospitalizations. This aligns with previous cross‐sectional US study, which reported that patients receiving eculizumab and ravulizumab, respectively, had an average of 0.57 and 1.36 hospitalizations in the prior 12 months [10]. Taken together, these findings may indicate a continued dependence on healthcare resources among patients receiving C5i, further contributing to the burden of disease.

Although the introduction of complement inhibitors have demonstrated favorable treatment outcomes, as noted by a recent systematic literature review and meta‐analysis [29], it is evident that significant challenges and unmet needs exist. In this study, a proportion of patients receiving C5i continued to demonstrate disease burden, including suboptimal laboratory parameters, persistent symptoms, and impaired quality of life. These findings highlight the importance of comprehensive disease assessment that integrates laboratory measures with patient‐reported outcomes, particularly as new therapeutic options emerge. Several novel therapies which target the complement pathway are being investigated in ongoing clinical trials [30, 31, 32]. In addition, the first‐in‐class oral monotherapy, iptacopan, has been approved in Europe, Japan, China, Korea, the United States, and Canada for the treatment of PNH [33, 34, 35, 36, 37, 38]. As shown by several studies, iptacopan has the potential to address these unmet needs, providing improved efficacy (comprehensive hemolysis control with the added benefit of fewer transfusions, and improvement in fatigue score and overall HRQoL) without sacrificing safety, beyond that achieved with C5i therapy [39, 40, 41]. Therefore, new complement inhibitors could further expand treatment options for patients with PNH. Evidence from real‐world studies, combined with clinical experience, will support identifying patients suitable for different complement inhibitors in PNH, enabling personalized care and addressing unmet needs.

### 4.1 Limitations

The DSP has several limitations. While minimal inclusion criteria governed the selection of participating physicians; participation was influenced by willingness to complete the survey. The DSP captures data from a consulting patient population, therefore patients who consult more frequently, such as those who are more severe or have additional complications, may have been more likely to be included. Responses may be subject to recall bias, a common limitation of surveys; the data were collected at the time of each patient's consultation and physicians had access to medical records to limit this. Despite such limitations, real‐world studies play an important role in highlighting areas of concern that are not addressed in clinical trials, including the burden of disease from both the physician and patient perspective.

## 5 Conclusion

This real‐world study demonstrated that, despite treatment with C5i, a proportion of patients with PNH remained anemic, experienced substantial fatigue, and reported impaired HRQoL. Many patients continued to require hospitalization and blood transfusions, indicating persistent clinical burden and continued use of healthcare resources. While C5i have improved outcomes for many patients, these findings highlight residual unmet needs and underscore the importance of comprehensive assessment, particularly as additional therapeutic options continue to emerge.

## Author Contributions

Conception or design of the work: Shreyans Gandhi, Markus P. Radsak, Yasutaka Ueda, Maria‐Magdalena Balp, Anggie Wiyani, Olivier Somenzi, Josefin Snellman, Yasmin Taylor, Alice Simons, and Jens Panse. Analysis or interpretation of data: Alice Simons and Yasmin Taylor. Manuscript drafting: Alice Simons, Yasmin Taylor, Maria‐Magdalena Balp, Anggie Wiyani, Olivier Somenzi, and Josefin Snellman. Manuscript revision: Shreyans Gandhi, Markus P. Radsak, Yasutaka Ueda, Maria‐Magdalena Balp, Anggie Wiyani, Olivier Somenzi, Josefin Snellman, Yasmin Taylor, Alice Simons, and Jens Panse. All authors have read and approved the final manuscript and agree to be accountable for all aspects of the work.

## Conflicts of Interest

Shreyans Gandhi has received travel support and honoraria from Alexion, SOBI, Gilead, Celgene and Jazz; consultancy fees from Alexion, SOBI, Pfizer, and Novartis; and institutional research funding from Alexion. Markus P. Radsak has received travel support from SOBI, AOP, AbbVie, and Johnson & Johnson; consultancy fees from Pfizer, Novartis, SOBI, MSD, Lilly, GSK, Alexion, Merck, Beigene, BMS, Johnson & Johnson, AOP, AstraZeneca, AbbVie, Cogent, and RYVU; and research funding from the Foundation “Litchterzellen”, EKFS, and DFG. Yasutaka Ueda has received honoraria from Alexion, Chugai, Incyte, Kaken, Nippon Shinyaku, Novartis, Sanofi, and Sobi; consultancy fees from Alexion, Asahi Kasei, Chugai, Janssen, Novartis, Ono, Sanofi and Sobi; research funding from Chugai. Maria‐Magdalena Balp, Olivier Somenzi, Josefin Snellman are employees of Novartis Pharma AG, Basel, Switzerland. Anggie Wiyani is employee of Novartis Pharmaceuticals UK Ltd, London, UK. Yasmin Taylor is an employee of Adelphi Real World, of which Alice Simons is a former employee. Jens Panse has received consultancy fees, honoraria including travel support from Alexion, Astra Zeneca, Blueprint Medicines, Boehringer Ingelheim, Bristol Myers Squipp, F Hoffmann La Roche, MSD, Novartis, Omeros, Pfizer, Sanofi, and SOBI; and research funding (to the institution) from Alexion, Blueprint Medicines, Cogent, F Hoffmann La Roche, Novartis, and Omeros

## Disclosure

Data collection was undertaken by Adelphi Real World as part of an independent survey entitled the Adelphi PNH DSP. The DSP is a wholly owned Adelphi Real World product, of which Novartis were one of multiple subscribers. Novartis did not influence the original survey through either contribution to the design of questionnaires or data collection. Publication of survey results was not contingent on the subscriber's approval or censorship of the manuscript.

## Ethics Statement

The DSP complies with all relevant market research guidelines and legal obligations. Physician‐reported data were collected according to European Pharmaceutical Marketing Research guidelines [42] and received ethical exemption from the Pearl Institutional Review Board (#21‐ADRW‐127). The DSP is non‐interventional and employs cross‐sectional data collection with elements of retrospective chart review, and no identifiable protected health information was extracted during the study. Data were anonymized and aggregated for analysis and publication, thus collected in such a way that patients and physicians could not be directly identified.

## Consent

Written informed consent was collected from all participants prior to data collection via tick boxes within the surveys.

## Supporting information



Supporting File: 1

## Data Availability

The data that support the findings of this study are available from the corresponding author upon reasonable request.
