# Supplementary material for: Clinical Profile, Humanistic and Economic Burden of Paroxysmal Nocturnal Hemoglobinuria in Patients Treated With C5 Inhibitors
Source: EJHaem. 2026 Feb 18;7(1):e70248. doi: 10.1002/jha2.70248 (PMC12916017; doi:10.1002/jha2.70248)
Supplement: Supplementary file 1 — Supporting File: 1 [file JHA2-7-e70248-s001.docx]

Supplementary Table 1. Comorbidities of C5i-treated patients prior to onset of PNH symptoms, after onset of PNH symptoms, and after diagnosis of PNH

|  | **Overall (n=288)** | **Prior to onset of PNH symptoms** | **After onset of PNH symptoms** | **After diagnosis of PNH** |
| --- | --- | --- | --- | --- |
| **Comorbidities, n (%)** |  |  |  |  |
| None | 97 (33.7) | - | - | - |
| Hypertension | 62 (21.5) | 46 (74.2) | 2 (3.2) | 14 (22.6) |
| Anxiety | 35 (12.2) | 26 (74.3) | 4 (11.4) | 5 (14.3) |
| Aplastic anemia | 33 (11.5) | 20 (60.6) | 10 (30.3) | 3 (9.1) |
| Depression | 30 (10.4) | 22 (73.3) | 2 (6.7) | 6 (20.0) |
| Diabetes | 30 (10.4) | 27 (90.0) | 0 (0.0) | 3 (10.0) |
| Renal disease/failure | 25 (8.7) | 11 (44.0) | 9 (36.0) | 5 (20.0) |
| Gastroesophageal reflux disease | 23 (8.0) | 18 (78.3) | 3 (13.0) | 2 (8.7) |
| Peptic ulcer disease | 19 (6.6) | 14 (73.7) | 3 (15.8) | 2 (10.5) |
| Migraine | 18 (6.3) | 12 (66.7) | 4 (22.2) | 2 (11.1) |
| Myelodysplastic syndrome | 15 (5.2) | 8 (53.3) | 6 (40.0) | 1 (6.7) |
| Congestive heart failure | 14 (4.9) | 9 (64.3) | 1 (7.1) | 4 (28.6) |
| Hypoplastic anemia | 14 (4.9) | 4 (28.6) | 6 (42.9) | 4 (28.6) |
| Chronic pulmonary disease | 11 (3.8) | 10 (90.9) | 0 (0.0) | 1 (9.1) |
| Rheumatologic disease | 11 (3.8) | 9 (81.8) | 2 (18.2) | 0 (0.0) |
| Mild liver disease | 11 (3.8) | 3 (27.3) | 3 (27.3) | 5 (45.5) |
| Myocardial infarction | 10 (3.5) | 7 (70.0) | 1 (10.0) | 2 (20.0) |
| Other | 10 (3.5) | 7 (70.0) | 0 (0.0 | 3 (30.0) |
| Peripheral vascular disease | 9 (3.1) | 4 (44.4) | 3 (33.3) | 2 (22.2) |
| Cerebrovascular disease | 6 (2.1) | 1 (16.7) | 2 (33.3) | 3 (50.0) |
| Ulcerative colitis | 6 (2.1) | 5 (83.3) | 0 (0.0) | 1 (16.7) |
| Crohn’s disease | 5 (1.7) | 4 (80.0) | 1 (20.0) | 0 (0.0) |
| Dementia | 3 (1.0) | 1 (33.3) | 0 (0.0) | 2 (66.7) |
| Moderate or severe liver disease | 2 (0.7) | 2 (100.0) | 0 (0.0) | 0 (0.0) |
| Any malignancy, including leukemia and lymphoma | 1 (0.3) | 0 (0.0) | 0 (0.0) | 1 (100.0) |
| Lupus anticoagulant/Anti-phospholipoid syndrome | 1 (0.3) | 1 (100.0) | 0 (0.0) | 0 (0.0) |
| Parkinson’s disease | 1 (0.3) | 1 (100.0) | 0 (0.0) | 0 (0.0) |

C5i, C5 inhibitor; PNH, paroxysmal nocturnal hemoglobinuria
